# Exploring the Methodological Benefits and Challenges of Utilising a Photovoice Methodology With Individuals in Recovery From Problem Substance Use

**DOI:** 10.1177/10497323231217601

**Published:** 2024-01-11

**Authors:** Emma Smith, Melody Carter, Elaine Walklet, Paul Hazell

**Affiliations:** 1 Institute of Psychiatry and Neuroscience, 4616 Kings College London, London, UK; 2 School of Nursing and Midwifery, 8709 University of Worcester, Worcester, UK; 3 School of Psychology, 2376 University of Gloucestershire, Cheltenham, UK; 4 School of the Arts, 8709 University of Worcester, Worcester, UK

**Keywords:** Photovoice, visual research, participatory action research, substance use, recovery

## Abstract

Photovoice is a type of visual research method which supports participants to reflect upon their experiences by capturing digital images. It is a methodology that is routinely used with groups that could be considered vulnerable, as a way of allowing participants to tell their stories for themselves. This article details the process of conducting a Photovoice study with individuals in recovery from problem substance use and reflects on the methodological benefits and challenges of utilising a visual research methodology with this population. Researchers wishing to conduct a Photovoice study with individuals in recovery should be mindful of striking a delicate balance between respecting an individual’s autonomy and ensuring their wellbeing. Although ethically complex, Photovoice is an ideal method for research with this population as it allows participants to convey meaning and introduce narratives for themselves in an engaging way.

Photovoice has been described as a type of participatory action research (PAR) method which asks individuals to record their experiences through photographs (Wang & Burris, 1997). Researchers subsequently interview participants regarding their community experiences, with photographs taken by participants used during interviews as catalysts for discussion (Wang & Burris, 1997). In a PAR project, all steps are done with community/participant involvement to ensure that data is authentic to individual experiences. However, the degree to which the community is involved may differ significantly from one project to another (Greene, 2013).

‘Power’ is a crucial underlying tenet of PAR, since the goal is to support participant empowerment through contribution to the research process (Baum et al., 2006). Labonte defines empowerment as the dynamic shifting of power quality relations between people such that the relationship bends towards equity by reducing inequalities and power differences (Labonte, 1990). PAR studies such as Photovoice are thus done in an effort to empower, promote voices, and support the dialogues of participants within themselves, as well as within their wider community (Freire, 1970).

In 2020, seven clients from a harm reduction organisation in the Southwest of England were recruited to participate in a Photovoice study to understand the individual experience of substance use recovery (Smith et al., 2022). Data collection for this study included a group training workshop, a group meeting, individual interviews (conducted over the telephone due to the COVID-19 pandemic), and a final group workshop.

Drawing on experience from this Photovoice study, this article aims to highlight the difficulties and strengths of conducting a Photovoice study with individuals in recovery from problem substance use while also providing recommendations for future researchers who wish to conduct visual research with this population. This paper also seeks to examine the often-contentious understanding of Photovoice as an example of PAR by interrogating the choices made regarding participant decision-making and group dynamics within an exemplar research study. Ethical approval for this study was provided by the Ethics Committee of the University of Worcester (approval number: CHLES181900369-R). All patients provided written informed consent prior to study enrolment.

## Existing Methodological Discussions and Evaluations of Photovoice

There is much debate in the Photovoice literature regarding the optimal way to successfully implement a Photovoice study. Following recruitment, the routine structure for most Photovoice studies consists of a training session where Photovoice is introduced to the group, cameras distributed, and an initial photographic theme posed. This is usually followed by allowing participants time to take photographs, meeting to discuss/share their progress, followed by sharing the photographs with a wider audience (Sutton-Brown, 2014). The empirical study in question followed these steps, with slight deviations due both to the COVID-19 pandemic and the stated wish of participants.

In this section, we provide an overview of the literature within several domains of interest while also describing how these were implemented by the exemplar Photovoice study.

### Participant Training and the Researcher’s Role

As Photovoice uses pictures as a primary tool for data collection, it is important that participants understand what Photovoice is as well as basic camera usage. Previous systematic reviews have found that some form of initial Photovoice training for participants was included in all assessed studies (Chinn & Balota, 2023; Hergenrather et al., 2010; Suprapto et al., 2020). Most of these training sessions consisted of discussions concerning the goals of the project, what Photovoice is, and some form of training with cameras while also collecting informed consent for participation. The empirical study in question followed these outlined steps. There was one initial mandatory workshop for participants facilitated by the lead researcher where training occurred.

Photovoice emphasises the content of the photograph and the meanings attributed to them and not necessarily photographic quality, so camera training generally only covers basic camera functions (Harley, 2012; Sutton-Brown, 2014). In his review of visual participatory research methods, Packard asserts that it is potentially problematic for the researcher to adopt the role of ‘teacher’ in the field and that unequal power dynamics are immediately and irrevocably established the moment the researcher provides instruction for equipment operation (Packard, 2008). This is especially true for marginalised and/or vulnerable populations whose primary currency is knowledge related (i.e. street smarts) (Packard, 2008). Many participants may not feel comfortable asking for camera instructions because they consider camera use to be ubiquitous, and when knowledge is someone’s primary form of capital, admitting ignorance renders one powerless. For this reason, Packard decided not to offer any camera training for participants (homeless individuals) out of concern that it would increase existing power division. He also stressed the importance of low cost cameras because it increases the probability that the participant will be familiar with the technology (Packard, 2008).

While Packard’s approach differs from the standardised method of photography training described by Wang and Burris (1994), it demonstrates the importance of choosing methodological approaches which build upon the initial state of participants’ knowledge (Bergold & Thomas, 2012; Rosen & Painter, 2019). Each approach intends to accomplish separate things and will ultimately affect the outcome of studies. Projects which choose to incorporate a workshop element should give participants time to practise with the cameras, ask how experienced participants are with cameras, and be prepared to check in during the photoshoot period to assess progress (Bendell & Sylvestre, 2017; Mamary et al., 2007).

### Participant Decision-Making Power

To generate meaningful participation in Photovoice studies, it is important that developmental work is not simply imposed on passive recipients. It is therefore crucial that participants in PAR projects take an active role in the decision-making process (Gabrielsson et al., 2022). This is an important step in attending to the complicated power dynamics, unequal access to resources, and differing stakes in the research process which must be addressed in order to facilitate a successful PAR project (Worthington et al., 2016). Much of this will depend on how the method is applied.

For the empirical study in question, the research plan was for participant decision-making and group dynamics to be placed at the centre of the process. This included allowing participants to decide how long the project would last (specifically how many interviews they wanted to do), what theme to focus on, and whether they would continue the project as a group or as individuals. At this time, participants decided they wished to have individual interviews to share their photographs, a decision that was contradictory to emancipatory ideals of PAR which stress group dialogue and the co-creation of knowledge (Freire, 1970).

This impacted data collection as the lack of sharing photographs within a group likely impeded any interactive group discourse where participants would have an opportunity to comment or reconstruct narratives related to the photographs of other group members. However, participants made this decision citing the privacy concerns of sharing their photographs and stories with the group. Participants ultimately met up twice more as a group, once to do a check-in together about how the Photovoice process was going and again during a final workshop to choose photographs for dissemination and feedback on preliminary analysis.

### Focus of Photovoice Photographs

The participant training is generally followed by a photoshoot period prior to participants sharing their photographs to the group/researcher. To assist participants in focusing on the research question, guidance is regularly given to prompt picture taking (Anderson et al., 2023; Hergenrather et al., 2010). In some instances, this guidance is minimal so as to encourage participants to photograph without undue researcher interference (Ronzi et al., 2016). Additionally, Sutton-Brown suggested that encouraging participants to define and conceptualise the research question for themselves enables the supposition of agency within the research process (Sutton-Brown, 2014).

In some cases, more detailed prompts are offered to participants to assist them in the photo taking process, particularly if participants struggle hypothesising project themes independently. At the initial workshop for this empirical research study, participants were invited to choose a theme to focus five photographs on. The theme ultimately agreed upon was ‘people, places, or things meaningful to you’. A person’s identity is constructed of more than just their group membership, so it is important to ask participants to focus on prompts which capture many aspects of their lives, and which do not deconstruct them to group membership alone.

The overall narrative produced by Photovoice projects will be driven equally by what participants choose to depict in their photographs and what they choose to exclude. During interviews, it is suggested that researchers ask questions related not only to why an individual took a specific picture but to also direct lines of inquiry to things participants wanted to take pictures of but did not (Rose, 2016). This practice creates a participatory and conversant process, which allows the data to reflect the context the participant was aiming to demonstrate. It is important that researchers are sensitive to the needs of their population of interest and design the logistical elements of their studies with this in mind.

### Dissemination of Photographs

If appropriate, photographs taken for a Photovoice study are routinely shared with a wider audience after participants have had a chance to share them with other participants and the researcher. During the final group workshop, participants were able to feed back to the lead researcher that they were interested in sharing their photographs with the general public to increase understanding of their experiences. As the research took place during the COVID-19 pandemic, the participants wished for their photographs to be shared on an outdoor mural wall so that the photographs could reach audience members safely in a way that directly communicated with their community about their experiences.

What makes Photovoice different from other visual research methods is the emphasis on socio-political change in which the newly ‘empowered’ engage with their communities and connect with policy makers to enact change (Hergenrather et al., 2010; Johnston, 2016). Wang and Burris emphasised community and social change during the inception of Photovoice in the hopes that the images produced and the ensuing discussion would act as a tool to reach, inform, and organise community members (Wang & Burris, 1997).

Photographic dissemination can be accomplished in several ways, the most common being a photography exhibition, as was the case with the empirical study in question (Bendell & Sylvestre, 2017; Hergenrather et al., 2010). Other forms of dissemination include community forums for policy makers, presentation projects, letter writing campaigns, research action plans, and one-on-one viewing sessions (Bendell & Sylvestre, 2017; Brazg et al., 2011; Hergenrather et al., 2010; Johnston, 2016; Mizock et al., 2014). Despite the central focus on social action and policy change, there is often vagueness regarding how projects enact this, leading to a somewhat romanticised view of participatory photography and its potential to attain transformational results (Johnston, 2016).

Three systematic reviews of the Photovoice literature have indicated that there has been little attempt from Photovoice projects to evaluate any substantive long-term impact of the method on individuals or communities, stating a lack of consistent reporting (Catalani & Minkler, 2010; Han & Oliffe, 2016; Hergenrather et al., 2010). Additionally, a recent systematic review of international healthcare literature found that Photovoice rarely translated into positive physical/mental health outcomes nor long-term community functions (Halvorsrud et al., 2022). Unfortunately, very few Photovoice studies influence policy, something which is ethically problematic when policy influence is frequently reported as a primary goal of Photovoice (Golden, 2020).

Previous literature has compared Photovoice projects which contain blanket claims of empowerment to ‘parachute projects’ or other one-time research interventions with the potential to raise false hopes when efforts to rally public support or inform public policy are unsuccessful (Higgins, 2016; Johnston, 2016). Wang and Burris themselves have commented that for a Photovoice project to be effective, a positive and engaged audience open to unconventional ideas is necessary (Wang & Burris, 1994). This has led some academics to question why it is that finding a solution to social issues is often the responsibility of those most affected by them (Mitchell, 2011).

Some Photovoice studies advocate for arranging dissemination strategies with potential partners early, while others advocate for organising with policy makers later in the project while being conscious of not unrealistically raising participants’ expectations (Cantarero-Arévalo & Werremeyer, 2021; Johnston, 2016; Wang et al., 2000). Gaining preliminary support from policy makers may help to foster an environment of support rather than challenging policy makers with photographic issues (Goodhart et al., 2006). However, others have suggested that this contradicts Photovoice’s commitment to a participant-guided agenda and that researchers are prematurely establishing project aims and influencing participant representation by making dissemination decisions at the onset (Creighton et al., 2018; Sutton-Brown, 2014).

### Ethical Issues Specific to Photovoice

Ethical issues in Photovoice primarily revolve around informed consent, visual representation, power, copyright, and confidentiality/anonymity (Catalani & Minkler, 2010; Cox et al., 2014; Creighton et al., 2018; Hannes & Parylo, 2014; Wang & Redwood-Jones, 2001).

Special considerations regarding informed consent are presented by Photovoice projects due to their tendency to work with marginalised members of a population. Given the vulnerability of participants, many researchers consider ethical approval as a starting point and are continuously mindful of ethical issues (Horsfall et al., 2018). In accordance with Economic and Social Resource Council guidelines, consent is seen as an ongoing, open-ended, and voluntary process not simply resolved through the formal signing of a consent document (ESRC, 2010).

Photovoice projects often present unique challenges related to subjects who appear in photographs and ensuring that consent is collected from them (Ronzi et al., 2016). Although photography is generally considered an innocent act, it is possible that appearing in photographs of marginalised individuals may unfairly identify individuals or generate suspicion amongst community members (Prins, 2010). To overcome this, most Photovoice projects suggest crafting an informed consent form to give to individuals who appear in photographs (Hannes & Parylo, 2014; Rose, 2016).

Photovoice research also runs the risk of being ethically problematic through the reproduction of oppressive power relations (Higgins, 2016). Asking participants to document the realities of communities has the potential to be disempowering if it is not done with a focus on giving individuals a voice to speak about issues important to them (Abma et al., 2022; Cook & Buck, 2010). Photovoice aims to level the power imbalances inherent in traditional qualitative research. However, power imbalances will always exist, and power can never be distributed entirely equally between participants and researchers. This is a consequence of differing access to resources and divergent stakes in the process and outcome of research. It is important that these complicated power dynamics are acknowledged rather than ignored and that clear boundaries are discussed. Communication in a Photovoice context should not refer to the act of a researcher exploring for information, but rather a two-way dialogical process.

Another important consideration in Photovoice projects concerns copyright and ownership of photographs taken during the study. Most Photovoice projects accept the default UK legal position that photos should be considered the property of the photographer (participant) and that all participating individuals should receive personal copies of their photographs (Cook & Buck, 2010; Rose, 2016). However, collaborating institutions may have their own individualised protocol when it comes to issues of copyright, and this should be considered when planning a Photovoice project. Additionally, with digital photograph sharing and the internet, it is possible that photographs may be shared beyond what the participant expects or wishes (Creighton et al., 2018). It is important that researchers acknowledge these risks and that participants are given decision-making power over where their photographs are ultimately distributed.

Issues of anonymity and confidentiality also abound in Photovoice studies. There can be no guarantees of complete anonymisation of data for dissemination purposes as the totality of this task is fundamentally impossible, particularly when photographs are shared publicly. Photovoice has historically taken place with small, marginalised community groups, and for this reason, it may be simple for those familiar with a community to identity participants (Chapman et al., 2017). This could result in discomfiture on behalf of participants and result in damage to reputation, personal relationships, loss of employment, or criminal prosecution. Considering this, some academics maintain that anonymous photos may be the safest option, particularly for individuals who use drugs who are at potential risk of additional stigma if identified (Carlberg-Racich, 2021). However, this is not always a straightforward process.

For example, in Creighton and colleagues’ work reflecting on a Photovoice study which focused on family members who had been bereaved through male suicide, they describe a research participant who decided to share a self-portrait for the study (Creighton et al., 2018). The study facilitators worried about the potential implications of identification in this way, worrying that the participant’s mind may change over time or that her grief could impede her ability to give full, informed consent. However, they ultimately decided to include her self-portrait as it was the stated wish of the participant and seemed antithetical to exclude it within a methodology that centred on giving voice to participants’ experiences.

Within the context of Photovoice, it is imperative that researchers have the ability to make appropriate ethical decisions that operate in the best interests of research participants within the context of a specific study (Wiles et al., 2008). The development of trust is essential and should be of the upmost priority. It is also essential that participants decide themselves how and where their images are shared, and researchers must continuously strike the delicate balance between upholding both the privacy and agency of participants. This will differ according to how participants wish to share their images and may vary between individuals with distinct privacy considerations.

Additionally, the law makes it clear that confidentiality is not absolute where there is a public interest to disclose, but it is also apparent that it is not a legal duty (although in some cases it may be a professional one). Confidence should be maintained in circumstances which could positively inform public knowledge, even if this regards illegal activity. However, confidentiality should be breached in instances where participants report the endangerment of themselves or another person (Denzin & Lincoln, 2011; Surmiak, 2020). Participants should be explicitly informed of the boundaries of confidentiality.

## Recommendations for Using Photovoice for Individuals in Recovery: Challenges and Benefits

Several challenges and benefits of utilising a Photovoice methodology with individuals in recovery from substance use are discussed below. Key recommendations for future researchers who wish to undertake visual research with individuals in recovery from problem substance use can be found at the bottom of each subsection.

### Methodological Challenges

#### Informed Consent Concerns

One of the most important elements of a Photovoice project is the collection of written informed consent for individuals appearing in participants’ photographs (Creighton et al., 2018; Harley, 2012; Johnston, 2016; Wang & Redwood-Jones, 2001). During the initial participant workshop, a discussion occurred explaining the informed consent form for individuals appearing in images to ensure that they provided explicit permission. Several members of the group expressed anxiety around approaching people to appear in photographs, and so strategies to overcome this were discussed.

It became clear that some participants interpreted this as a discussion of ways people could appear in photographs without providing informed consent (e.g. taking photographs from a distance or obscuring an individual’s face). It was explained to participants that because of the sensitive nature of the project, it was especially important that people knew they were appearing in photographs, and participants responded to this idea positively.

Participants also had questions around the potential implications of identifying themselves as someone in recovery when they were asking others to appear in their photographs. One participant suggested the possibility of not truthfully identifying themselves as someone in recovery which led to a group discussion regarding the implications of appearing in a photography project of this nature without knowledge or consent. It was stressed to participants that they should not approach anyone to appear in their photographs if they did not feel comfortable doing so or explaining what the study was about.

Additionally, this study contended with negotiating informed consent from deceased individuals. For example, one participant was pregnant at the time of her participation and had recently lost her ex-partner to suicide. One of the reasons that she wanted to participate in the study was to share his story in a dignified and humanising way. For her, this meant taking a photograph of the image used on his funeral flyer, where her ex-partner’s face was clearly visible. The decision was made by the research team that the participant should reach out to his family to have them sign the informed consent form for his image to be used in the study. However, she was advised to forgo this option if contacting them would be upsetting.

Ultimately, this participant did reach out to her ex-partner’s family and was able to get their written informed consent for his image to be disseminated. This ultimately proved to be appropriate as when the images were displayed publicly on an outdoor mural wall, a family member of the ex-partner responded to an audience survey. This person reported that it was emotional and moving to see their loved one displayed in the exhibition and overall reacted positively to the inclusion of his image. However, if informed consent had not been sought from his family, it likely could have been upsetting to see an image of a deceased loved one in public unexpectedly.

Another example of an issue with informed consent related to the inclusion of an image within an image. One participant photographed a wall of pictures displayed in the room of his dry house, including one of his young niece with her face clearly visible. The research team had not anticipated an instance where a minor’s face would be visible in a photograph nor one where someone’s image was captured unintentionally. Although this participant collected informed consent for other individuals who appeared in his photographs, it was clear he did not consider it necessary for this photograph as it was an image of different photographs. Ultimately, the research team decided to blur the face of the child so that the image could be included in dissemination. This was a potentially contentious choice as there is debate in the Photovoice literature between obfuscating meaning and protecting anonymity (Creighton et al., 2018; Evans-Agnew & Rosemberg, 2016; Rose, 2016).

*Key take away: *Researchers should remain ethically reflexive regarding the informed consent process as it is impossible to consider all unique scenarios that may present themselves. However, it is important that participants have a clear understanding of the reason for collecting informed consent for Photovoice studies focused on sensitive subjects and that participants’ wellbeing and the right to anonymity are at the centre of decisions*.

#### Participants’ Desire Not to Use Pseudonyms

During the initial workshop, participants asked the lead researcher if they could use their real names for photographic dissemination. During the subsequent group meeting, participants were asked why most of the sample (five of eight participants) did not want to use a pseudonym. The reasons listed included a wish to be transparent, not being embarrassed, an aspiration to share their stories, and a desire to reduce stigma. They also mentioned a desire to create recovery awareness, indicating that they were hoping to exhibit these photographs to the general public and that they would be proud to be directly attributed for their work.

The research team were left with a conundrum. The choice was to honour participants’ stated wishes and direction in line with the ethos of a participatory-driven project or to centre their right to confidentiality. This was particularly salient considering the sensitive subject matter that participants were photographing. It was determined by the research team that the real names of participants would not be presented to view in the main text. This was decided because of the potential implications (both known and unknown) of sharing names that may not have been immediately clear to participants (Wang & Redwood-Jones, 2001; Worthington et al., 2016).

However, this was a potentially disenfranchising decision, as other literature argues that it is paternalistic for researchers to impose their own judgements in determining what is right for participants, particularly within a research methodology that advocates for participant involvement in decision-making (Creighton et al., 2018; Nykiforuk & Vallianatos, 2018; Sitter, 2017a). Additionally, more recent Photovoice literature has discussed the ways in which the focus on preserving anonymity could stifle and disempower participants, impacting the ability for studies to include their stated wishes (Becot et al., 2023). While privacy is a key component in any Photovoice study and the decision to use real names should be justifiable, the stated wish of participants is also paramount, and upon reflection it was counterintuitive and invalidating for the empirical study to disregard this (Evans-Agnew & Rosemberg, 2016).

*Key take away: *Decide from onset if participants will use real names or pseudonyms. This will partially depend on the population of interest and their unique considerations as well as the stated goals of the project. All decisions should be defensible with participants’ wellbeing and best interest in mind. Researchers should be consistently mindful of the potential for removing agency from research participants by making decisions for them*.

#### Possible Triggers Due to Participation

Another challenge of this research project concerned the omnipresence of triggering situations. The focus for this study resulted in some participants choosing to take images of things they considered meaningful when they were problematically using substances. In some instances, this meant placing themselves in potentially triggering situations to complete the project as requested. This is a particularly salient concern for individuals in recovery from problematic substance use, specifically the use of alcohol, as individuals in recovery must contend with the environmental risks presented by its persistent availability and marketing (Shortt et al., 2017). For example, one participant took an image of a supermarket wine aisle because of the significance it had on his life and the lengths he now takes to avoid it. However, to do this meant placing himself in a potentially triggering situation to capture an image which demonstrated his experiences.

Considering this, the relationship between triggers, relapse, and the potential risk this poses to individuals in recovery should be considered when seeking ethical approval for future visual research studies. However, it is impossible for a research team to know what exactly will be triggering or upsetting for specific individual participants prior to their agreement to participate (Rhynas et al., 2020). Rather than telling them what will/will not be triggering for them, it is suggested that the researchers simply inform participants that this risk exists, and they should be made aware of it so that they may make this distinction for themselves. Participant safety is a key ethical concern in a Photovoice study, and this extends to psychological safety as well as the physical safety of participants (Evans-Agnew & Rosemberg, 2016).

*Key take away*: Researchers should consider the potential consequences of participants revisiting meaningful places (both physical and emotional), and participants should be made aware of this risk, although it is not the researcher’s responsibility to inform them what they can and cannot photograph.*

### Methodological Benefits

#### Photovoice as a Recruitment Tool

When asked why participants wanted to initially participate, the most common answer was to occupy their time and minds. The use of photography seemed to motivate people with a creative interest to participate when they may not have been otherwise interested. Likewise, the use of photographs seemed to attract participants who were interested in learning and practicing photography. This highlights the appeal of participatory documentary photography as a potentially more interesting form of data collection compared to traditional research (Coemans & Hannes, 2017; Prins, 2010; Rose, 2016).

For several participants, the Photovoice study appeared to represent an opportunity for community integration and individualised support, not unlike other community activities they were engaging with during recovery (Kaplan et al., 2012; Rhynas et al., 2020; Salzar, 2006). Some participants also engaged as they thought it would be a good way to showcase their recovery by demonstrating involvement in something ‘positive’. There existed the perception that participation was a meaningful activity, much of this due to the preconception that photography itself is a worthwhile and significant activity to engage with.

It was stressed to participants during the Photovoice workshop and within the informed consent process that this study was academic in nature and should not be misconstrued as a therapeutic endeavour (Elmir et al., 2011; Jansen, 2020; Wang & Redwood-Jones, 2001). Nevertheless, some participants appeared to find the interview to be a cathartic experience which gave them the opportunity to discuss important and meaningful topics, an additional factor which may have motivated their desire to participate.

*Key take away: *The use of photography works as an effective research tool as it can be perceived as an interesting and novel way of capturing information. The possibilities inherent in this should be amplified in terms of recruitment and engagement with a Photovoice study.*

#### Participants Enjoyment of the Photovoice Process

Participants expressed gratitude to the lead researcher for facilitating the study and reported enjoying the Photovoice process, saying that it helped them to reflect upon experiences in a novel way. Some also conveyed that they would be interested in following the project into the future and to continue working as a group, indicating the important role that group membership played in their overall experience (Bergold & Thomas, 2012; Robeyns, 2005).

Others expressed particular interest in the potential to share the images with the general public. This desire to share images publicly (and in doing so share their story with a wider audience) indicates a desire to implement some form of community/social action within the Photovoice project. However, it is important to differentiate between the enjoyment participants may feel completing a Photovoice study and sharing images from making grandiose claims regarding social action implementation (Higgins, 2016; Fairey, 2018; Golden, 2020).

This critique is not to discount the tangible benefit that partaking in Photovoice may or may not have afforded participants. Although not a measure of social action, the continued engagement in sharing their story is an indication of the desire to be involved with community-oriented initiatives (Gabrielsson et al., 2022). For example, one participant found that his participation was a largely positive thing, primarily because it gave him something to do and focus on during the COVID-19 lockdown which had impacted his involvement with other community activities. Other participants expressed a similar desire to continue with photography and reported that the Photovoice project had inspired them to reinvest their time into creative endeavours. This is particularly true for individuals in recovery who often seek group and/or community activities they enjoy to occupy their newfound time (Rhynas et al., 2020). Unfortunately, the COVID-19 pandemic meant that avenues for group connection within the empirical study could not be fully explored, and it is likely that additional group discourse would have expanded participants’ positive perception of Photovoice.

*Key take away: *Participants reported enjoying Photovoice, particularly the group comradery and reflective components. Future researchers should consider engagement opportunities for the participant group beyond the scope of the study (if possible) and differentiate between participants’ enjoyment of the study and claims of social action*.

#### Participant-Driven Research Method

One of the clear benefits of Photovoice in this context was that it permitted the interview to transition in a quick but in-depth way through multiple topics related to mental health, substance use, and recovery. This allowed participants the ability to control their own narratives in a sensitive way, adding an additional layer of complexity and interest to the research encounter (Glaw et al., 2017). While it is impossible to know what participants may have spoken about without the use of photographs, participants made use of images to introduce multiple topics covering several different aspects of an individual’s life and recovery experience. However, it also generates the consideration that this only allowed the researcher to conduct a ‘surface level’ exploration of factors an individual considered important in their recovery.

This may have been due to the theme participants chose to photograph (‘people, places, and things meaningful in recovery’). When participants selected this, it was hoped that a broad theme would allow participants to photograph individually important aspects of their lives. However, it is also possible that this wide-ranging theme left participants with a lack of direction. It is very likely that a more specific theme would have generated different photographs, perhaps allowing for a more in-depth look at specific aspects of the recovery experience (Najib Balbale et al., 2014).

Photovoice was used to explore experiences and community membership in a way that may not have been possible with other less participatory-driven methodologies. This is partially because of the questions asked during the interview, but also because of what participants were able to volunteer as they presented their narratives. The application of Photovoice allowed multiple subjects to be broached in a relatively short amount of time in a sensitive and thought-provoking way. It is also possible that, without the use of photographs, certain topics would not have been broached by participants as they were able to control the direction of the interview through the photographs they shared.

Photovoice was particularly beneficial as photographs constituted a useful icebreaker, leaving participants feeling at ease and in control of the research encounter. One reason for this may have been because some participants in this study expressed a prior interest in photography which helped engage them with the project. The use of photography as a beneficial tool in interview scenarios has been discussed previously, and methodological findings from this current work support these arguments (Rose, 2016; Coemans & Hannes, 2017; Creighton et al., 2018).

*Key take away: *Photographs can work in research as a useful methodological tool, particularly when they increase participants’ narrative control. Future researchers should consider the research question they are attempting to answer in terms of the prompt provided to participants to ensure images taken for the study are relevant and topical*.

## Discussion

Conducting a Photovoice study with individuals in recovery from problem substance use was an ethically challenging process that resulted in narrative-rich data that is useful in understanding the complexity of individual experiences. Past literature has described the methodology as a type of participatory action research method, yet for this to be true participants would had to have major input in developing the study, from design and recruitment onwards (Fricas, 2022; Baker & Wang, 2006; Catalini & Minkler, 2010; Wang & Burris, 1997). In Sitter’s work critiquing the notion that Photovoice is a type of PAR, she points out that the methodologies differ widely in terms of researcher positionality, decision making power, and the length of time devoted during the study period (Sitter, 2017b).

It must be acknowledged that the empirical study in question, while attempting to utilise a participatory framework, did not accomplish this goal. To be a true example of PAR, this study would have needed to better integrate participant decision-making into all elements of study design. This ultimately impacted how the Photovoice methodology was utilised in this context, although it was not dissimilar from the design of other Photovoice studies (Hergenrather et al., 2010). This questions the narrative presented by some Photovoice studies that social action and empowerment are an achievable end goal of Photovoice participation, as a participatory design centred around decision-making is often difficult to meaningfully actualise (Desyllas, 2014; Mitchell et al., 2005; Padilla et al., 2018; Rosen et al., 2011; Wang & Burris, 1994).

To help achieve this, researchers must be consistently reflexive regarding the challenge of respecting participant autonomy and voice while simultaneously allowing them to drive the narrative of the project in a way that brings personal meaning from engagement. This may include setting ground rules regarding what will and will not be shared in terms of ethically complex material. This is particularly important if there is a public dissemination component to the Photovoice study. Additionally, Photovoice studies which focus on sensitive research topics may consider adding clear caveats to the ‘informed consent to appear in photographs’ form so that individuals may explicitly consent to their photographs appearing in certain dissemination categories but not others (e.g. in a thesis but not online).

This adds to the argument in support of informed consent documents for individuals appearing in photographs but also takes this forward in arguing that these documents should include additional detail and allow individuals to clearly specify which specific mediums they consent for their image to be shared within (Rose, 2016; Wang & Redwood-Jones, 2001). Future research should also consider different methods of ethically obtaining informed consent for individuals who are deceased and with whom the participant is not related.

This research study also contended with the potential of participants becoming emotionally triggered from participation. Although the risk of emotional distress is possible with many qualitative studies dealing with sensitive topics, it is even more acute when asking participants to revisit (often physical rather than metaphysical) spaces. This is particularly salient for individuals in recovery from problematic alcohol use (Shortt et al., 2017). Future studies should consider the potential risks involved in participants revisiting triggers and ensure that participants are aware of this so that participation does not put them at increased risk of emotional distress. At the same time, researchers should be mindful of being paternalistic with participants and not assume what might be emotionally difficult for them to revisit through their photographs.

Despite these ethical challenges, participants in this Photovoice study reported enjoyment and stated that participating provided them with a fresh outlook on recovery. It is not uncommon for Photovoice projects to report findings such as these. Previous Photovoice literature has reported that participants enjoy the process of taking photographs, being part of something positive, creating awareness, and having an opportunity to both hear others’ stories and to tell their own (Anderson, et al., 2023; Mamary, et al., 2007; Rosen, et al., 2011; Thompson et al., 2008).

While Photovoice may be perceived as a more interesting and engaging research method which has the potential to inspire these feelings, it is important to interrogate why participants report these types of responses. Future researchers should be mindful of making grandiose claims regarding the impact of Photovoice studies. However, findings of enjoyment and engagement should not be ignored, and it is possible that recovery practitioners may find Photovoice to be a useful exercise to stimulate conversation and engage with clients.

Projects which seek to engage with stakeholders from the beginning may have a clearer focus on social action. However, the participants in this current project primarily sought to share photographs and raise awareness, hoping to use their voices to create understanding and empathy which may not always translate to clear social action outcomes (Purtle & Roman, 2015). Future studies should consider the social action aim they wish to achieve from the onset of a project. This may mean that participants are recruited for a longer period to ensure that their viewpoints are fully integrated and implemented within the study design. If participant groups wish to enact purposeful action within their communities, it is the responsibility of the research team to liaise with stakeholders and policymakers as soon as possible to engage them during several different study time periods (Lofton & Grant, 2021).
